# Antibiotic Resistance in *Neisseria gonorrhoeae*: Challenges in Research and Treatment

**DOI:** 10.3390/microorganisms10091699

**Published:** 2022-08-24

**Authors:** Boris Shaskolskiy, Ilya Kandinov, Ekaterina Dementieva, Dmitry Gryadunov

**Affiliations:** Center for Precision Genome Editing and Genetic Technologies for Biomedicine, Engelhardt Institute of Molecular Biology, Russian Academy of Sciences, 119991 Moscow, Russia

Gonococcal infection caused by the Gram-negative bacteria *Neisseria gonorrhoeae* is one of the most common sexually transmitted infections (STIs) worldwide. The public health importance of gonorrhoea is defined by several aspects, such as the continued high morbidity rate, predominant distribution among reproductive-age individuals with a pronounced negative impact on fertility, increased risk of co-infection with other STIs, absence of a gonococcal vaccine, and the progressive resistance of *N. gonorrhoeae* to antimicrobial drugs. At present, third-generation cephalosporins (ceftriaxone), together with azithromycin, are used as a combined antimicrobial therapy for the treatment of gonorrhoea in most countries around the world [1]. In December 2020, the U.S. Centers for Disease Control and Prevention issued updated treatment guidelines for gonococcal infection, increasing a single intramuscular (IM) dose of ceftriaxone from 250 mg to 500 mg for uncomplicated urogenital gonorrhoea [2]. It is expected that the updated guidelines will enable patients to be cured in a shorter period of time, which may slow down the rate of gonococcal infection [3]. The European guidelines, updated in 2020 [4], recommend dual antimicrobial therapy with 1 g ceftriaxone (IM) plus a single 2 g oral dose of azithromycin, or ceftriaxone 1 g (IM) alone in settings where in vitro antimicrobial susceptibility testing has shown an absence of ceftriaxone resistance; test of cure is mandatory, and a doxycycline regimen is administered if *Chlamydia trachomatis* infection has not been excluded [4].

Effective and accessible antimicrobial treatments are essential for the management of gonorrhoea; however, antimicrobial resistance in *N. gonorrhoeae* has emerged to all previous first-line therapeutic drugs, such as sulfonamides (the first resistant isolates appeared in the 1930s), penicillins (in the 1940s), tetracyclines and early-generation macrolides (in the 1960s), spectinomycin (in the 1970s), fluoroquinolones and azithromycin (in the 1990s), and third-generation cephalosporins (in the 2000s) [5]. Sporadic gonococcal isolates with ceftriaxone resistance have been described in many countries [6], and the first global failure with ceftriaxone plus azithromycin therapy was verified in the United Kingdom [7]. This case was associated with the internationally spreading multidrug-resistant and ceftriaxone-resistant (minimum inhibitory concentration, MIC_CRO_ = 0.5 mg/L) isolate belonging to the FC428 clade, initially described in Japan in 2015, which is further evolving [5,7]. In general, the resistance of *N. gonorrhoeae* to ceftriaxone is a rare phenomenon, even though the first isolates resistant to ceftriaxone—Japanese H041 (MIC_CRO_ = 2 mg/L) [8] and French F89 (MIC_CRO_ = 1 mg/L) [9]—appeared in 2011 and 2012, respectively. In 2019, in the European Union, ceftriaxone-resistant isolates were detected in two urogenital cases: one in Belgium (MIC_CRO_ = 0.5 mg/L) and one in Portugal (MIC_CRO_ = 0.25 mg/L) [10].

The decrease in ceftriaxone susceptibility (i.e., increase in the MIC value of the drug) in *N. gonorrhoeae* is caused by two groups of mechanisms. The first group comprises mutations in the targets of cephalosporin action, such as penicillin-binding proteins (PBPs) encoded by *penA* and *ponA* genes [11,12]. The second group comprises mutations leading to the impaired influx and increased efflux of antibiotics into and out of the cell (*porB* and *mtrR* genes). In most cases, resistance to cephalosporins is associated with the mosaic structure of the *penA* gene, which arose via the recombination of *penA* genes of commensal *Neisseria* species such as *N. perflava*, *N. sicca*, *N. polysaccharea*, *N. cinerea*, and *N. flavescens* [13]. Mutations in mosaic alleles leading to cephalosporin resistance include Ala311Val, Ile312Met, Val316Thr, Val316Pro, Thr483Ser, Ala501Pro, Ala501Val, Asn512Tyr, and Gly545Ser substitutions in the PBP-2 penicillin-binding protein [14]. However, mutations causing resistance in non-mosaic alleles have also been identified, i.e., PBP2: Ala501Val/Thr/Pro, Gly542Ser, Pro551Ser/Leu, which suggests that these substitutions resulted from the selection of gonococci rather than transfers from other species [11,15,16]. The primary determinant, associated with the high-level ceftriaxone resistance (MIC_CRO_ of 1–2 mg/L), is A501P substitution in mosaic *penA* allele, whereas A501T or A501V make a smaller contribution to the increase in MIC_CRO_ [9,11]. The main mutation in PBP1 leading to decreased susceptibility is Leu421Pro substitution [9,13]. Mutations in the promoter region of the *mtrR* gene include the deletion of a single adenine in the 13 bp inverted repeat sequence; mutations in PorB include substitutions in residues 120–121 [12,13].

An important modern trend is the development of regression models predicting the MIC values of antimicrobials through established genetic determinants of resistance. Corresponding models have been developed and validated to predict the *N. gonorrhoeae* ceftriaxone susceptibility level (MIC_CRO_) [17,18,19]. The greatest contributions to the increase in MIC_CRO_ were shown to be PBP2: Ala501Pro, Ala311Val, and Gly545Ser substitutions, Asp (345–346) insertion, and PorB: Gly120Arg substitution. However, the importance of the contribution of other determinants, such as the deletion of adenine (35delA) in the promoter region of the *mtrR* gene responsible for the change in MtrCDE efflux pump expression, remains to be clarified.

Despite the situation with resistance to ceftriaxone being stable, there is concern that isolates with the p*bla*_TEM-135_ plasmid may become widespread in China and worldwide [20]. The presence of plasmid-mediated β-lactamases in gonococci causes a significant increase in the level of resistance to penicillins (MIC_PEN_ ≥ 16 mg/L) compared with that related to chromosomal mutations (MIC_PEN_ = 0.12–1.0 mg/L) [5,21]. Similarly, an emergence of plasmid-mediated extended-spectrum β-lactamases that can hydrolyze cephalosporins may lead to a severalfold increase in the ceftriaxone MIC. Just a single Gly238Ser mutation in the *bla*_TEM-135_ gene will result in the *bla* gene variant encoding the TEM-20 β-lactamase which can hydrolyse both penicillins and cephalosporins. Although *N. gonorrhoeae* clinical isolates carrying the p*bla*_TEM-20_ plasmid have not been found in nature, this single change can lead to a very high level of ceftriaxone resistance (MIC_CRO_ ≥ 4 mg/L) [22], which could end the therapeutic use of third-generation cephalosporins. However, it is likely that these concerns are exaggerated, because growth curves of the genetically engineered *N. gonorrhoeae* strains with different p*bla*_TEM_ plasmids predicted a substantially reduced viability of the strain carrying the p*bla*_TEM-20_ plasmid compared with that of strains with the p*bla*_TEM-1_ or p*bla*_TEM-135_ plasmids [22].

Although gonococci with reduced susceptibility to cephalosporins occur sporadically in the global population (0.1–2%), the number of azithromycin-resistant *N. gonorrhoeae* isolates increases every year. In some European and Asian countries, the proportion of azithromycin-resistant isolates exceeds 5%, which is a threshold value for excluding this drug from therapy regimens, according to WHO recommendations [10,23,24,25]. For example, this situation has been observed in Guangzhou, China, where the proportion of azithromycin-resistant isolates increased from 8.60% to 20.03% between 2013 and 2020 [25].

To predict the resistance of *N. gonorrhoeae* to azithromycin, it is necessary to take into account that the *N. gonorrhoeae* genome may include up to four copies of the *rrn* operon (*rrnA*, *rrnB*, *rrnC*, and *rrnD*), which comprises the nucleotide sequence encoding 23S rRNA. It should particularly be noted that the level of resistance is also determined by the number of mutant alleles of 23S rRNA. Isolates with three or all four mutant alleles have been shown to be highly resistant (MIC_AZM_ > 256 mg/L), whereas isolates with only one copy of the 23S rRNA mutant allele exhibit an insignificant level of resistance, below or at the threshold level [26]. The resistance of *N. gonorrhoeae* to macrolides is also mediated by *mtrR* and/or *mtrD* gene polymorphisms associated with its mosaic structure, which lead to the activation of the MtrCDE efflux system and, consequently, the withdrawal of azithromycin from the cell [27]. Another factor influencing the resistance of *N. gonorrhoeae* to macrolides is the synthesis of 23S rRNA methyltransferases ErmA, ErmB, ErmC, and ErmF. These enzymes provide the dimethylation of adenine A2058 in the peptidyltransferase loop of the V-domain of 23S rRNA, which leads to modification of the target of antibiotic action, reductions in binding affinity, and the formation of resistance [28].

Notably, the widespread distribution of a particular genetic line is not always associated with antimicrobial resistance. Different gonococcal genetic lines, which can be defined as molecular types (*Neisseria gonorrhoeae* multiantigen sequence typing (NG-MAST)), may have different survival strategies. Thus, analysis of NG-MAST types predominant worldwide—225, 1407, 2400, 2992, and 4186—and to genogroup 807, the most common genogroup in Russia, revealed differences in antimicrobial resistance profiles. The multidrug-resistant isolates belonging to NG-MAST types 225, 1407, and 2400 possessed gonococcal genetic islands (GGIs) without lesions in the genes necessary for functional activity of the corresponding type IV secretion system. On the other hand, more susceptible isolates from genogroup 807 and NG-MAST types 2992 and 4186 either lacked a GGI or carried critical mutations in genes essential for DNA secretion [29]. T4SS encoded by genes located on a GGI allows the bacterial cell to produce and secrete single-stranded DNA, which can then be specifically recognized by pili on recipient cells via DNA uptake sequences and recombined into the genome. The association between the presence of a GGI and genotype-predicted resistance to numerous antibiotics, including cefixime, ciprofloxacin, and penicillin, has been observed previously [30]. However, simply the presence of a GGI, which are found in ~80% of *N. gonorrhoeae* isolates, does not guarantee its proper function for ssDNA secretion, and lesions/changes in the key genes that could interfere with the entire T4SS system must be considered [29]. In-depth analyses of the significance of such differences in the ongoing evolution of *N. gonorrhoeae* are undoubtedly required.

The persistence of the population of drug-resistant *N. gonorrhoeae* isolates requires a search for new targets of antimicrobials, such as virulence factors [31]; the development and validation of new antimicrobials, such as zoliflodacin, gepotidacin or solithromycin [32,33,34]; and a thorough evaluation of the use of existing classes of antimicrobials. The latter includes the uses of ciprofloxacin, gentamicin, and ertapenem in selected therapy regimens according to European guidelines for the diagnosis and treatment of gonorrhoea in adults [4]. However, their large-scale introduction should be possible only after a detailed study of the relationship between the genetic background and a level of phenotypic resistance. An example of a prospective clinical study of single-dose oral ciprofloxacin treatment in patients with *N. gonorrhoeae* infections was recently described [35]. A 100% cure rate was observed in those subjects who were culture-positive at enrolment with the wild-type gyrA serine 91 *N. gonorrhoeae* genotype. Such studies are important for the safe reintroduction of fluoroqionolones for the treatment of gonorrhoea.

Due to the ability of *N. gonorrhoeae* to acquire genetically determined resistance to all antimicrobials, emergence of a form of the gonococcal infection that does not respond to therapy may be a serious threat. The antigenic plasticity of the pathogen, coupled with its complex relationship with the human immune system, poses significant challenges in the development of a fully effective vaccine against gonorrhoea. However, there have been several recent advances that support the feasibility of gonococcal vaccine development [36]. One observational study suggested that a vaccine against the closely related bacteria *N. meningitidis*, the outer membrane vesicle (OMV) meningococcal B vaccine MeNZB, had an effectiveness of 31% against infection with *N. gonorrhoeae* [37]. A newer four-component meningococcal B vaccine, 4CMenB (marketed as Bexsero), which contains the MeNZB OMV component plus three recombinant protein antigens, has been shown to induce cross-reactive antibodies to *N. gonorrhoeae* proteins, including the Neisseria heparin binding antigen (NHBA) [38]. The use of optimized NHBA alone, or in combination with other antigens, proved its ability to induce antibodies in mice that promote complement activation and mediate bacterial killing via both serum bactericidal activity and opsonophagocytic activity [39].

A recent major challenge is an increasing number of cases of urethritis caused by *N. meningitidis*, another microorganism of the *Neisseria* genus [40,41]. An emerging non-groupable *N. meningitidis* urethrotropic clade is also similar to *N. gonorrhoeae*, in that the isolates are capable of nitrite-dependent anaerobic growth [42]. This phenotype corresponds to the presence of gonococcal-like *aniA* and *norB* genes, which catalyse the conversion of nitrite to nitrous oxide. However, this phenomenon cannot be considered a result of the acquisition of *aniA* and *norB* genes by *N. meningitidis* isolates, because they are present in both species. Nitrite-dependent anaerobic growth is due to the phase variation mechanism, as one of the factors of adaptation of *N. meningitidis* isolates to a gonococci ‘lifestyle’. On the other hand, whole-genome sequencing of the *N. meningitidis* urethrotropic clade revealed 581 recombination events, of which 138 were inferred to have originated from *N. gonorrhoeae* [40]. The transfer of antimicrobial resistance determinants is of particular concern. Confirming this risk, one isolate had a gonococcal-like *mtrR* sequence associated with elevated azithromycin MIC of 2 mg/L [40]. Another study reported an *N. meningitidis* strain with *N. gonorrhoeae* penicillin-resistance-associated *penA* alleles from an invasive meningococcal disease case involving a patient on long-term complement inhibitor therapy and daily penicillin chemoprophylaxis [43]. Furthermore, Deghmane et al. reported the emergence of meningococcal clinical isolates with decreased susceptibility to third-generation cephalosporins [44]. Sequence analysis revealed a mosaic *penA* allele (*penA327)* identical to the *N. gonorrhoeae* penAXXXIV allele, which was previously found in the isolates with a decreased susceptibility to ceftriaxone. The acquisition of *penA327* by meningococci may have occurred from the gonococcal penAXXXIV allele during a mixed gonococcal/meningococcal urethral infection, further emphasizing the possibility of the sexual transmission of meningococci and the need to study the co-evolution of the two pathogens.

A possible solution to the problem of eradicating pathogens of the *Neisseria* genus may be hidden in the mechanisms of interspecies struggles. In recent years, several studies on the ability of *Neisseria* species to inhibit each other’s growth have been published. For example, *N. cinerea* inhibits the growth of *N. meningitidis* in epithelial cell culture [45], and the DNA secreted by *N. elongata* and several other nonpathogenic *Neisseria* species is capable of killing *N. gonorrhoeae* [46]. Aho et al. demonstrated the antimicrobial activity of *N. mucosa* against *N. gonorrhoeae* [47]; Custodio et al. found that the ability of *N. cinerea* to inhibit the growth of *N. meningitidis* and *N. gonorrhoeae* is due to a change in the expression of the type VI secretion system [48].

It is also pertinent to know that there is a close association between vaginal microbial flora dysbiosis and an increased incidence of urinary tract infections, including gonorrhoea. Thus, as an alternative to developing new antibiotics, one focus should be on developing new probiotics or live supplements, such as beneficial microbes that act against pathogens [49]. There are about 50 different species residing within the vagina, such as *Lactobacillus* species, that are regarded as regulators of the vaginal microenvironment. Through their production of lactic acid, lactobacilli may help maintain a low vaginal pH which can inhibit the growth of other bacteria. Colonization with hydrogen peroxide producers from *Lactobacillus* sp. is associated with a lower frequency of gonorrhoea [50]. Probiotics cannot be considered as a panacea in the treatment of gonorrhoea, but they can serve as a useful supplement to reassure compromised states of the urogenital system.

Recently, advanced findings regarding the diagnostics, antimicrobial susceptibility surveillance, treatment, and follow-up of gonorrhoea patients have proven essential in controlling gonococcal infection. Contemporary investigations using advanced genomic, proteomic, metabolomic, and bioinformatics approaches, and their results on the role of *N. gonorrhoeae* in pathogenesis, antimicrobial resistance, and epidemiology, are presented in this Special Issue. In addition, future research to clarify the *Neisseria* pathogen–host and commensal–host interactions as a tool for promising therapy development will be discussed by invited leading authors.
